# Patterns of Postmastectomy Radiotherapy in Immediate Breast Reconstruction—Results From the iBRA‐2 Cohort Study

**DOI:** 10.1155/ijbc/5902426

**Published:** 2026-05-18

**Authors:** Tim Rattay, Adam Trickey, Rachel L. O’Connell, Rajiv V. Dave, Joanna Skillman, Nicola L. P. Barnes, Matthew D. Gardiner, Chris Holcombe, Shelley Potter

**Affiliations:** ^1^ Leicester Cancer Research Centre, University of Leicester, Leicester, UK, le.ac.uk; ^2^ Population Health Sciences, University of Bristol, Bristol, UK, bristol.ac.uk; ^3^ Department of Breast Surgery, The Royal Marsden NHS Foundation Trust, Sutton, UK, nhs.uk; ^4^ Nightingale Breast Unit, Manchester University NHS Foundation Trust, Manchester, UK, mft.nhs.uk; ^5^ Department of Plastic Surgery, University Hospitals Coventry and Warwickshire NHS Trust, Coventry, UK, nhs.uk; ^6^ Nuffield Department of Orthopaedics, Rheumatology and Musculoskeletal Sciences, University of Oxford, Oxford, UK, ox.ac.uk; ^7^ Department of Plastic Surgery, Wexham Park Hospital, Slough, UK, nhs.uk; ^8^ Swansea Bay University Health Board, Swansea, UK; ^9^ Bristol Breast Care Centre, North Bristol NHS Trust, Bristol, UK, nhs.uk

**Keywords:** breast cancer, cohort study, immediate breast reconstruction, radiotherapy, trainee collaborative

## Abstract

**Purpose:**

Long‐term data indicate that postmastectomy radiotherapy (PMRT) is associated with improved overall survival in node‐positive breast cancer patients. Immediate breast reconstruction (IBR) remains controversial in the context of planned PMRT, but rates of IBR are increasing. The aim of this study was to examine patterns of PMRT in patients undergoing mastectomy with or without IBR.

**Methods:**

Data were collected from 2526 patients enrolled in the iBRA‐2 prospective cohort study undergoing 2606 mastectomies with and without IBR between 1 July 2016 and 31 December 2016. Patients were recruited consecutively at 71 centres across the United Kingdom (UK) and Ireland and at five international centres. Univariable and multivariable logistic regression models were used to explore associations between recommendation for PMRT and patient‐ and procedure‐related factors.

**Results:**

Of 2590 breast procedures included in the analysis, 696 were implant‐based, 105 pedicled‐flap and 230 free‐flap reconstructions, and 32.5% of implant‐based, 34.3% of pedicled‐flap and 35.7% of free‐flap reconstructions were recommended for PMRT. PMRT recommendation by cancer stage was 21% for T1‐2 N0, 65% for T1‐2 N1 and 89% for T_any_ N2 and T3 N_any_. In multivariable analyses, patients with invasive disease undergoing implant‐only reconstruction, but not pedicled‐flap or free‐flap reconstruction, were less likely to be recommended for PMRT than those undergoing mastectomy alone. The likelihood of being recommended for PMRT differed by region.

**Conclusion:**

Although IBR was more likely to be performed for lower‐stage cancers and in younger patients with fewer comorbidities, a third of patients undergoing IBR were recommended for PMRT. This adds to a growing body of evidence that IBR is becoming an acceptable option for women requiring PMRT. The study also highlighted regional variation in PMRT practice within the UK and Ireland, which merits further investigation.

## 1. Introduction

Breast cancer is the most common female cancer in Europe with 557,532 new cases diagnosed in 2022 [1]. Despite advances in treatment and early diagnosis, almost 40% of women undergo mastectomy as their primary surgery [2, 3]. After surgery, radiotherapy is the second most commonly used treatment for breast cancer. Historically, long‐term outcome data from randomised controlled trials have demonstrated that postmastectomy radiotherapy (PMRT) reduces recurrence rates and improves survival in all node‐positive breast cancer patients [4], and population‐based data have shown that use of PMRT in patients with 1–3 positive lymph nodes increased significantly between 2003 and 2012 [5, 6]. Several national treatment guidelines have recommended that radiotherapy be considered for all patients with node‐positive disease after mastectomy [7–9]. However, the role of PMRT in intermediate‐risk breast cancer (defined as pT1–2N1, pT3N0 or pT2N0 if also Grade 3 or with lymphovascular invasion) is less clear. Long‐term results of the Selective Use of Postoperative Radiotherapy After Mastectomy (SUPREMO) Medical Research Council (MRC) Phase III clinical trial have now been published and indicate that PMRT does not impact overall survival in patients with N1 disease (1–3 positive lymph nodes) or those with N0 disease and other high‐risk features [10].

Immediate breast reconstruction (IBR) is offered to women undergoing mastectomy with the aim of improving quality of life [11]. IBR remains controversial in the context of planned PMRT [12]. However, United States (US) Surveillance, Epidemiology, and End Results (SEER) data indicate that an increase in PMRT amongst women with Stage I–III breast cancer undergoing mastectomy did not lead to a concomitant decrease in IBR [6]. In fact, data from the US Mastectomy Reconstruction Outcomes Consortium (MROC) for > 2000 patients show that around 25% of implant‐based and half of autologous reconstructions received PMRT [13]. Following a review of 23 observational studies, the United Kingdom (UK) National Institute for Health and Care Excellence (NICE) concluded that there was insufficient evidence to indicate worse patient outcomes from PMRT in the setting of IBR and that IBR should be offered even if adjuvant RT is anticipated [14]. At the same time, rates of IBR and, in particular, implant‐only reconstruction have increased [15], yet there is a paucity of population‐based data on the use of PMRT in patients undergoing IBR in the UK and other European countries [16].

In reconstructive breast surgery, the trainee research collaborative model has emerged as a time‐ and cost‐effective method for delivering large‐scale prospective studies [17, 18]. The UK Breast Reconstruction Research Collaborative of breast and plastic surgeons has delivered the iBRA‐2 study to determine the impact of IBR on the delivery of adjuvant treatment [19]. The present study was undertaken to analyse patterns of PMRT amongst patients undergoing mastectomy with and without IBR enrolled in the iBRA‐2 cohort.

## 2. Methods

The methods of the iBRA‐2 cohort study have been reported elsewhere [19, 20]. Briefly, all breast and plastic surgical units performing mastectomy with and without IBR were invited to participate in the study via the professional associations (Association of Breast Surgery [ABS] and British Association of Plastic Reconstructive and Aesthetic Surgeons [BAPRAS]) and the breast and plastic surgery collaborative research networks (Reconstructive Surgery Trials Network [RSTN] and Mammary Fold Academic and Research Collaborative [MFAC]). Of 90 centres that expressed an initial interest, 76 centres enrolled 2526 consecutive patients between 1 July 2016 and 31 December 2016. The study sample covered approximately half of all UK breast units as well as five centres in Ireland (recruiting *n* = 103), four centres in Italy and one in Egypt (recruiting *n* = 145).

Consecutive women aged 18 or over undergoing mastectomy with or without IBR using any technique for invasive or preinvasive (ductal carcinoma in situ [DCIS]) breast cancer with curative intent were included. Patients were identified from multidisciplinary team (MDT) meetings, unit operating records and clinics. Patients were excluded if they were undergoing risk‐reducing surgery (without a therapeutic mastectomy for breast cancer), partial mastectomy including wide local excision with volume replacement (latissimus dorsi [LD] miniflaps, lateral intercostal perforator [LICAP] flaps or thoracodorsal artery perforator [TDAP] flaps) or displacement techniques (therapeutic mammaplasty) and if they had distant metastatic disease.

The iBRA‐2 study was classified as a service evaluation according to the NHS Health Research Authority online decision tool (http://www.hra-decisiontools.org.uk/research/), so ethical approval was not required. Each participating centre obtained local governance approvals prior to entering patients into the study. Data were collected prospectively, including baseline demographic and operative data. Oncological data and adjuvant treatment recommendations were collected from postoperative MDT meeting records. Pilot data collected at a number of participating sites between 1 May 2016 and 30 June 2016 suggested that adjuvant therapy was unlikely to commence earlier than 6 weeks postoperatively. Data collection in patients not requiring adjuvant treatment therefore continued from the last definitive cancer surgery until 6 weeks following surgery.

Study data were collected and managed using REDCap (Research Electronic Data Capture) electronic data capture tools hosted at the Kennedy Institute of Rheumatology, University of Oxford [21, 22]. REDCap is a secure, web‐based software platform designed to support data capture for research studies, providing (1) an intuitive interface for validated data capture; (2) audit trails for tracking data manipulation and export procedures; (3) automated export procedures for seamless data downloads to common statistical packages; and (4) procedures for data integration and interoperability with external sources. For quality assurance purposes, the lead investigator at each site was asked to independently validate 5%–10% of the data. If concordance between the data entered on REDCap and that independently validated was < 90%, the unit’s data were excluded from the analysis, consistent with the quality assurance procedure used in other collaborative projects.

### 2.1. Study Definitions and Endpoints

Primary and secondary outcomes in iBRA‐2 were selected based on current best practice [23] and national breast cancer guidelines [24]. The endpoint in the present study was the use of adjuvant radiotherapy, defined as the recorded postoperative MDT outcome ‘radiotherapy recommended’ or ‘radiotherapy to be discussed’. For analysis purposes, patients in iBRA‐2 were categorised into four groups according to the most complex surgical procedure received as follows: (i) mastectomy only (no reconstruction), (ii) mastectomy and IBR with implant‐only techniques, (iii) mastectomy and IBR with pedicled flaps and (iv) mastectomy and IBR with free‐flap techniques.

Implant‐based procedures included any reconstruction in which only expanders/implants were used to reconstruct the breast, either with or without biological (e.g., acellular dermal matrix) or synthetic (e.g., titanium‐coated polypropylene) mesh irrespective of whether the implant/expander was placed in a pre‐ or subpectoral position. Pedicled‐flap procedures included any pedicled flap used to reconstruct the breast with or without an implant/expander, including LD and transverse rectus abdominis myocutaneous (TRAM) flaps. Free‐flap procedures included any technique in which a microvascular free flap was used for IBR, including deep inferior epigastric perforator (DIEP), superficial inferior epigastric perforator (SIEA), superior and inferior gluteal artery perforator (SGAP and IGAP) and transverse upper gracilis (TUG) flaps. All complications were defined a priori [20]. Major complications were defined as any complication requiring readmission or reoperation. Minor complications were defined as those managed conservatively. An analysis of short‐term complications is described elsewhere [19].

### 2.2. Statistical Analysis

Data are presented per breast procedure, so any patient who fulfilled the inclusion criteria and had bilateral surgery was counted as two procedures. Descriptive summary statistics were calculated for each variable in the whole cohort and subdivided by procedure type (mastectomy only, implant‐only reconstruction, pedicled‐flap reconstruction and free‐flap reconstruction). Categorical data were summarised by counts and percentages, and continuous data were summarised by median, interquartile range (IQR) and range. Characteristics of procedure groups were compared using chi‐square tests for categorical variables and the Kruskal–Wallis test for continuous variables. Amongst patients with invasive disease, univariable and multivariable logistic regression models were used to explore associations between clinicopathological variables and use of PMRT, including patient‐ and procedure‐related factors, namely, age, body mass index (BMI), smoking status, American Society of Anaesthesiologists’ (ASA) grade, diabetes, ischaemic heart disease (IHD), other comorbidities, chemotherapy, oestrogen receptor (ER) status, human epidermal growth factor receptor 2 (HER‐2) status, lymphovascular invasion, tumour grade, multifocality and procedure type.

The correlation between proportions of patients undergoing PMRT and IBR by region was determined using Pearson’s *r* statistic. Whether different regions of the UK and Ireland had differing rates of recommending PMRT was assessed using multivariable logistic regression, adjusted for BMI, comorbidities, smoking status, ASA, ER and HER‐2 status, lymphovascular invasion, multifocality and tumour grade, with clustering by centre. This was performed separately for patients undergoing mastectomy only and those undergoing any IBR. All analyses were performed in STATA 19.5 (StataCorp, Texas).

## 3. Results

Of 2526 patients recruited into the iBRA‐2 study, 1528 underwent mastectomy only, 666 underwent implant‐only reconstruction, 105 received pedicled flaps and 227 underwent free‐flap reconstruction. As 80 patients had bilateral cancer surgery and there were 16 procedures with missing outcome data, a total of 2590 breast procedures were included in the analysis. On a per‐breast basis, Table 1 and Supporting Information 1: Table S1 summarise patient demographics and adjuvant therapy decisions by procedure type. Patients undergoing mastectomy alone (*n* = 1559 breast procedures) were older than those undergoing IBR and were more likely to have comorbidities as indicated by ASA grade. Patients undergoing IBR were more likely to have lower‐stage cancers. Adjuvant chemotherapy was more likely to be recommended after mastectomy only than after IBR. Adjuvant radiotherapy was recommended or for discussion in 46.0% of mastectomies, in 32.5% of implant‐based reconstructions and in 34.3% and 35.7% of pedicled‐flap and free‐flap reconstructions, respectively.

**Table 1:** Patient demographics and proportion of patients offered adjuvant radiotherapy by type of surgical procedure.

Per‐breast data	Mastectomy only		Implant reconstruction		Pedicled‐flap reconstruction		Free‐flap reconstruction		Total		*p* value
	(*n* = 1559, 60.2%)		(*n* = 696, 26.9%)		(*n* = 105, 4.0%)		(*n* = 230, 8.9%)		(*n* = 2590, 100%)		
Age (median, IQR)	65	54–75	50	43–57	52	47–60	50	45–56	58	48–69	< 0.001
ASA grade (*n*, %)											
1	339	21.7%	288	41.4%	40	38.1%	60	26.1%	727	28.1%	< 0.001
2	918	58.9%	385	55.3%	61	58.1%	161	70.0%	1525	58.9%	
3	288	18.5%	23	3.3%	3	2.9%	8	3.5%	322	12.4%	
4	6	0.4%	0	0.0%	0	0.0%	0	0.0%	6	0.2%	
Missing data	8	0.5%	0	0.0%	1	1.0%	1	0.4%	10	0.4%	
T stage (*n*, %)											
Tis	141	9.0%	162	23.3%	26	24.8%	57	24.8%	386	14.9%	< 0.001
T1‐2	1206	77.3%	472	67.8%	67	63.8%	142	61.7%	1887	72.9%	
T3	190	12.2%	54	7.8%	9	8.6%	18	7.8%	271	10.5%	
Missing data	22	1.4%	8	1.2%	3	2.9%	13	5.7%	46	1.8%	
N stage (*n*, %)											
N0	904	58.4%	517	75.3%	71	67.6%	163	70.9%	1655	63.9%	< 0.001
N1	391	23.9%	126	15.8%	16	15.2%	45	19.6%	578	22.3%	
N2	250	15.8%	42	5.9%	12	11.4%	14	6.1%	318	12.3%	
Missing data	14	1.9%	11	3.0%	6	5.7%	8	3.5%	39	1.5%	
Chemotherapy (*n*, %)											
Recommended	395	25.3%	154	22.1%	25	23.8%	39	17.0%	613	23.7%	0.001
For discussion	135	8.7%	37	5.3%	5	4.8%	9	3.9%	186	7.2%	
For Oncotype DX	93	6.0%	65	9.3	5	4.8%	17	7.4%	180	7.0%	
Received	236	15.1%	132	19.0%	21	20.0%	44	19.1%	433	16.7%	
Missing data	2	0.1%	0	0%	0	0%	0	0%	2	0.1%	
Radiotherapy (*n*, %)											
Recommended	631	40.5%	207	29.7%	35	33.3%	63	27.4%	936	36.1%	< 0.001
For discussion	86	5.5%	19	2.7%	1	1.0%	19	8.3%	125	4.8%	

### 3.1. PMRT Recommendation by Tumour and Nodal Stage

**Figure 1:**
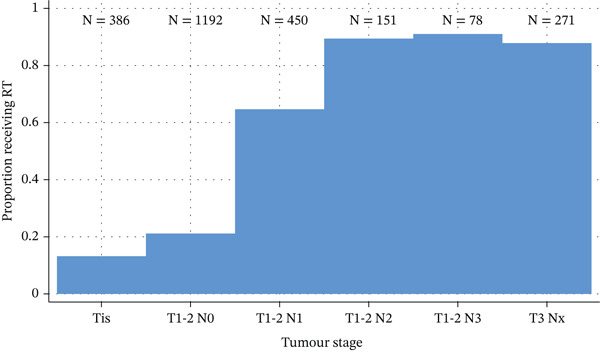
Proportion of patients (%, per‐breast data) recommended for postmastectomy radiotherapy by tumour stage.

Figure 1 shows the proportion of patients on a per‐breast basis (*n* = 2590) recommended for PMRT by tumour stage. Out of all patients with T3, N2 or N3 stage disease, 89% were recommended for radiotherapy, whilst 64.7% of patients with N1 stage disease were recommended for radiotherapy. Interestingly, 13.2% of Tis and 21.1% of N0 stage patients were also recommended for radiotherapy. When subdivided by tumour and nodal stage, there was little difference in PMRT recommendation percentages between surgical procedure types (Supporting Information 1: Table S2).

Table 2 and Supporting Information 1: Table S2 show the patient and tumour variables associated with PMRT recommendation for patients with invasive disease. In multivariable analysis, patients with larger tumours and more advanced lymph node stage were more likely to receive a recommendation for PMRT. Patients undergoing pedicled‐flap or free‐flap reconstruction were as likely as those having mastectomy to be recommended for PMRT. However, women receiving implant‐only reconstruction were less likely to be offered PMRT compared to patients undergoing mastectomy only (adjusted odds ratio 0.65, 95% confidence interval: 0.43–0.97, *p* = 0.035).

**Table 2:** Unadjusted and adjusted∗ odds ratios of PMRT recommendation in patients with invasive disease (*n* = 2204 breast procedures).

Per‐breast data	Univariable analysis			Multivariable analysis		
	Odds ratio	95% CI	*p* value	Adjusted odds ratio	95% CI	*p* value
Tumour size (invasive)						
0–9 mm	1	Comparator		1	Comparator	
10–20 mm	0.74	0.57–0.95	0.020	0.65	0.46–0.91	0.012
20–50 mm	1.91	1.48–2.46	< 0.001	1.18	0.84–1.67	0.340
> 50 mm	14.08	8.91–22.24	< 0.001	11.76	6.18–22.40	< 0.001
Lymph nodes						
N0	1	Comparator		1	Comparator	
N1	6.88	5.19–9.13	< 0.001	4.77	3.45–6.60	< 0.001
N2	27.19	17.43–42.40	< 0.001	16.34	9.31–28.69	< 0.001
Type of surgery						
Mastectomy only	1	Comparator		1	Comparator	
Implant	0.61	0.46–0.81	0.001	0.65	0.43–0.97	0.035
Pedicled flap	0.77	0.44–1.37	0.383	0.63	0.32–1.25	0.184
Free flap	0.80	0.53–1.21	0.297	0.74	0.38–1.45	0.379

Abbreviation: CI, confidence interval.

∗The multivariable analysis also included age, body mass index, comorbidities (ischaemic heart disease, diabetes and other), smoking status, chemotherapy recommendation, ASA grade, ER and HER‐2 status, lymphovascular invasion, multifocality and tumour grade. Full results are shown in Supporting Information 1: Table S2.

### 3.2. Regional Variation in PMRT

Excluding 145 patients from five centres outside the UK and Ireland, Supporting Information 2: Figure S1 shows no correlation between the proportion of patients recommended for PMRT and the proportion receiving IBR across the 14 regions (*r* = −0.23, *p* = 0.41).

Table 3 shows the proportion of patients recommended for PMRT subdivided by region, excluding centres outside the UK and Ireland. In adjusted analyses, women undergoing mastectomy in Northern Ireland/Ireland, the North of England, Yorkshire/Humberside, the West Midlands and Scotland were less likely to be recommended for PMRT than those in London and the Southeast. For patients undergoing IBR, the regional numbers were too small to subdivide further by procedure type. Nevertheless, women undergoing IBR in Yorkshire/Humberside were less likely to be recommended for PMRT than those in London and the Southeast of England.

**Table 3:** Adjusted∗ odds ratios of PMRT recommendation by region in patients undergoing mastectomy only and those undergoing IBR.

Per‐breast data	Mastectomy only (*n* = 1513)			Breast reconstruction (any) (*n* = 932)		
	Percentage RT recommended	aOR (95% CI)	*p* value	Percentage RT recommended	aOR (95% CI)	*p* value
London/Southeast	53%	1		39%	1	
East	48%	0.84 (0.46–1.52)	0.562	39%	0.71 (0.25–1.99)	0.512
East Midlands	49%	0.93 (0.46–1.89)	0.837	24%	0.45 (0.17–1.20)	0.110
Yorkshire/Humber	37%	0.49 (0.26–0.91)	0.024	24%	0.31 (0.11–0.83)	0.020
NI/Ireland	52%	0.30 (0.15–0.60)	0.001	47%	1.85 (0.65–5.28)	0.251
North	41%	0.43 (0.22–0.84)	0.014	25%	0.43 (0.17–1.05)	0.065
Scotland	44%	0.33 (0.17–0.64)	0.001	48%	1.32 (0.46–3.80)	0.613
Wales/Southwest	47%	0.74 (0.37–1.47)	0.387	34%	1.81 (0.61–5.35)	0.282
West Midlands	47%	0.51 (0.28–0.95)	0.034	37%	1.02 (0.23–4.50)	0.974

Abbreviations: aOR, adjusted odds ratio; CI, confidence interval; NI, Northern Ireland.

∗Multivariable analysis also included age, body mass index, comorbidities (ischaemic heart disease, diabetes and other), smoking status, chemotherapy recommendation, ASA grade, ER and HER‐2 status, lymphovascular invasion, multifocality, lymph node status and tumour size and grade. Full results are shown in Supporting Information 1: Table S3.

## 4. Discussion

The aim of this study was to analyse patterns of PMRT amongst patients undergoing mastectomy with and without IBR. The primary outcome and rates of postoperative complications in the iBRA‐2 patient cohort have been reported elsewhere [19]. In relation to PMRT, the present study found that between 32.5% and 35.7% of women undergoing IBR were recommended for or were for discussion of radiotherapy compared to 46% of those receiving mastectomy. These figures are in keeping with several previously published observational studies showing that between 29% and 43% of patients with IBR are irradiated [13, 18, 25, 26]. IBR was more likely to be performed for lower‐stage cancers and in younger patients with fewer comorbidities. Nevertheless, in patients with invasive disease, after adjusting for patient and tumour variables, only women undergoing implant‐only reconstruction were statistically less likely to be recommended for PMRT. This was not the case for those undergoing pedicled‐flap or free‐flap reconstruction. This adds to a growing body of evidence that IBR is becoming an acceptable option for women requiring PMRT [27–29] and that PMRT is only one of many factors in the decision for/against IBR, which include consideration of patient and oncological factors as well as surgeon and patient preferences. Indeed, it has been shown that breast cancer MDTs cannot predict with certainty whether a patient will require adjuvant PMRT [30].

There was no correlation between the proportion of patients recommended for PMRT and the proportion of IBR across different UK regions. This suggests that participating centres offered all types of IBR irrespective of whether PMRT was expected in the adjuvant setting, in keeping with findings from a similar US population‐based study [6] and current clinical guidelines [14]. The individual choice of IBR technique is likely to depend on several patient and surgical factors as well as locally available expertise and access to immediate free‐flap reconstruction. However, in patients requiring PMRT, implant‐only IBR may be associated with more complications compared to autologous IBR [13, 18]. Follow‐up data from two prospective cohort studies showed lower satisfaction amongst patients with irradiated implant‐only reconstruction [31, 32]. Nevertheless, further work is required to establish the impact of PMRT on the long‐term outcomes of IBR to provide high‐quality data to inform practice. Initial results from nonrandomised studies comparing neoadjuvant versus adjuvant radiotherapy in patients scheduled for IBR are promising and demonstrate the feasibility and safety of this approach [33], but the results of randomised trials will take some years to mature.

Compared to previous population‐based analyses showing between 30.3% and 40.5% of patients with N1 disease (1–3 positive lymph nodes) receiving PMRT before 2012 [5, 6], in our study, 64.7% of such patients as well as 87.8% of patients with T3N0 disease, representing 21.7% (total *n* = 562) of the study population, were recommended for PMRT. This confirmed the trend of a hitherto increasing number of patients with N1 and T3 disease being considered for PMRT [7, 8, 34]. Following publication of the SUPREMO trial [10], it remains to be seen if PMRT use for intermediate‐risk breast cancer (defined as pT1–2N1, pT3N0 or pT2N0 if also Grade 3 or with lymphovascular invasion) will reduce, as treatment recommendations for this group have recently been updated in national guidelines in the light of other recently published trials [9, 35]. Interestingly, the proportion of patients in our study with noninvasive cancer receiving PMRT (13.2%) appears relatively high compared to an earlier UK study [36], although our study was not restricted to the breast screening population, and the receipt of radiotherapy following mastectomy for DCIS may be related to margin status, grade and presence of microinvasion.

Subdividing the data from the UK regions and Ireland, we found that patients in Northern Ireland/Ireland, Scotland, the North of England and Yorkshire/Humberside were significantly less likely to be recommended for PMRT. This highlights some interesting geographical variation, which merits further investigation. A US‐based study found that the density of radiation oncology practices was associated with receipt of PMRT in patients with N1 stage breast cancer [5].

There is accumulating evidence that individual patient and tumour molecular profiles will be able to predict the response to adjuvant treatment. Several Phase III trials have reported on the effectiveness of the 70‐ or 21‐gene recurrence scores in selecting patients for adjuvant chemotherapy [37–39]. Genomic‐adjusted radiation dose (GARD) therapy may allow personalisation of radiotherapy on the basis of the biological effect of a given physical dose of radiation, calculated using individual tumour genomics, and has been shown to predict time to recurrence and overall survival [40]. In terms of complications from IBR, predictive risk models have been validated in the US with receipt of PMRT (or not) as a predictor [41, 42]. Other risk prediction models for individual radiotherapy side effects in the breast have been validated [43, 44], though none in the setting of IBR. In the future, personalised medicine approaches including the patient germline and tumour genomic profile may aid decision‐making around IBR and PMRT.

## 4.1. Strengths and Limitations

To our knowledge, this is the largest cohort study to date in the UK and Ireland investigating patterns of PMRT in patients undergoing mastectomy with and without IBR, but there are several limitations. Due to its observational design, the study is at risk of bias. Patients were recruited consecutively from participating centres, but there were baseline differences between the treatment groups. Although we adjusted for known confounders such as cancer stage, patient age, BMI, smoking status and ASA grade and excluded noninvasive disease from some of our analyses, we acknowledge that it is not possible to adjust for all potential confounders. Our study included patients from 71 centres in the UK and Ireland as well as five centres in Italy and Egypt. It is possible that participating units differed from those not taking part. However, approximately half of all UK breast units were represented in the study.

Due to the short follow‐up period (up to initiation of first adjuvant treatment), we were only able to analyse treatment recommendations and were unable to ascertain successful receipt or completion of PMRT in all patients. It was not possible to follow patients up to completion of adjuvant therapy with this surgical trainee collaborative design, but collaborations with oncology trainees should allow us to address this issue in the future. Moreover, a data linkage study is planned to explore long‐term oncological outcomes in terms of local recurrence, disease‐free survival and overall survival. Whilst it is not possible to establish causation with this observational study design, the design and conduct of randomised controlled trials in the setting of IBR are perceived as difficult due to patient and surgeon preferences for type and timing of breast reconstruction [26].

## 4.2. Conclusions

Although IBR was more likely to be performed for lower‐stage cancers and in younger patients with fewer comorbidities, approximately one‐third of patients in this population‐based cohort undergoing IBR were recommended for PMRT. About two‐thirds of all patients with T1‐2 N1 disease were recommended for PMRT, which reflects clinical treatment guidelines for this patient group at the time of study recruitment, and the present study provides an estimate of patients potentially eligible for treatment de‐escalation. Within the UK and Ireland, the study has also highlighted regional variation in PMRT practice that merits further investigation. Further work is ongoing to establish the impact of PMRT on the long‐term outcomes of different types of IBR to help women and surgeons make more informed decisions about breast reconstruction options.

## Author Contributions

S.P. and T.R. conceived the study design (subgroup analysis). T.R. contributed to the design, conduct of the study, analysis of the data and interpretation of the results and wrote the first draft of the paper. A.T. performed the analysis, contributed to data interpretation and drafted the manuscript. R.L.O.C., T.R., R.V.D., N.L.P. B., C.H. and S.P. contributed to the study design for iBRA‐2. M.D.G. and J.S. contributed to the study design and interpretation of the data.

## Funding

Funding was provided by the NIHR Leicester Biomedical Research Centre (10.13039/501100020013), the NIHR Bristol Biomedical Research Centre (10.13039/100015250) and the National Institute for Health Research (10.13039/501100000272; CL‐2017‐11‐02, DRF‐2014‐07‐079 and CS‐2016‐16‐019).

## Disclosure

A previous version of this paper was posted as a preprint on Research Square at https://www.researchsquare.com/article/rs-1108079/v1. All authors read and approved the final manuscript. The views expressed in this publication are those of the authors and not necessarily those of the NHS, the NIHR or the Department of Health and Social Care.

## Conflicts of Interest

T.R. is supported by the National Institute for Health Research (NIHR) Leicester Biomedical Research Centre and was previously an NIHR Clinical Lecturer (CL‐2017‐11‐02) and NIHR Doctoral Research Fellow (DRF‐2014‐07‐079). S.P. is supported by the NIHR Bristol Biomedical Research Centre and was previously an NIHR Clinician Scientist (CS‐2016‐16‐019). The other authors have no competing interests to declare.

## Supporting Information

Additional supporting information can be found online in the Supporting Information section.

## Supporting information


**Supporting Information 1** Table S1: Further patient demographics by type of surgical procedure. Table S2: Percentages where RT is recommended by MDT (or discussed with the patient) versus not recommended, stratified by procedure type and T stage or N stage. Table S3: Adjusted odds ratios for whether RT is recommended by MDT (or discussed with the patient) versus not recommended amongst (a) those undergoing mastectomy only and (b) those undergoing any other type of breast reconstruction.

**Supporting Information 2** Figure S1: Scatter plot of implant‐only reconstruction (IBR) percentages and postmastectomy radiotherapy (PMRT) recommendation percentages (*n* = 2381, excluding centres outside the UK and Ireland).

## Data Availability

The datasets generated during and/or analysed in the current study are available from the corresponding author on reasonable request.
