# Supplementary material for: Patterns of Postmastectomy Radiotherapy in Immediate Breast Reconstruction—Results From the iBRA‐2 Cohort Study
Source: Int J Breast Cancer. 2026 May 18;2026:5902426. doi: 10.1155/ijbc/5902426 (PMC13181802; doi:10.1155/ijbc/5902426)
Supplement: Supplementary file 1 — Supporting Information 1 Table S1: Further patient demographics by type of surgical procedure. Table S2: Percentages where RT is recommended by MDT (or discussed with the patient) versus not recommended, stratified by procedure type and T stage or N stage. Table S3: Adjusted odds ratios for whether RT is recommended by MDT (or discussed with the patient) versus not recommended amongst (a) those undergoing mastectomy only and (b) those undergoing any other type of breast reconstruction. [file IJBC-2026-5902426-s001.docx]

# Supplementary Material

Supp Table 1. Further patient demographics by type of surgical procedure.

| **Per breast data** | **Mastectomy only** | | **Implant reconstruction** | | **Pedicled flap reconstruction** | | **Free flap reconstruction** | | **Total** | | **p-value** |
| --- | --- | --- | --- | --- | --- | --- | --- | --- | --- | --- | --- |
|  | **(n=1559, 60.2%)** | | **(n=696, 26.9%)** | | **(n=105, 4.0%)** | | **(n=230, 8.9%)** | | **(n=2590, 100%)** | |  |
| **BMI** |  |  |  |  |  |  |  |  |  |  | <0.001 |
| Underweight | 34 | 2.2% | 18 | 2.6% | 0 | 0.0% | 2 | 0.9% | 54 | 2.1% |  |
| Normal | 456 | 29.3% | 338 | 48.6% | 37 | 35.2% | 69 | 30.0% | 900 | 34.8% |  |
| Overweight | 464 | 29.8% | 196 | 28.2% | 35 | 33.3% | 86 | 37.4% | 781 | 30.2% |  |
| Obese | 253 | 16.2% | 69 | 9.9% | 22 | 21.0% | 44 | 19.1% | 388 | 15.0% |  |
| Severely obese | 228 | 14.6% | 37 | 5.3% | 5 | 4.8% | 16 | 7.0% | 286 | 11.0% |  |
| Not reported | 124 | 8.0% | 38 | 5.5% | 6 | 5.7% | 13 | 5.7% | 181 | 7.0% |  |
| **Smoking status** |  |  |  |  |  |  |  |  |  |  | 0.019 |
| Non-smoker | 1104 | 70.8% | 515 | 74.0% | 75 | 71.4% | 164 | 71.3% | 1858 | 71.7% |  |
| Current smoker | 243 | 15.6% | 95 | 13.7% | 18 | 17.1% | 51 | 22.2% | 407 | 15.7% |  |
| Ex-smoker | 183 | 11.7% | 74 | 10.6% | 12 | 11.4% | 11 | 4.8% | 280 | 10.8% |  |
| Missing | 29 | 1.9% | 12 | 1.7% | 0 | 0.0% | 4 | 1.7% | 45 | 1.7% |  |
| **Co-morbidities** |  |  |  |  |  |  |  |  |  |  |  |
| Diabetes | 197 | 12.6% | 25 | 3.6% | 7 | 6.7% | 11 | 4.8% | 240 | 9.3% | <0.001 |
| IHD | 139 | 8.9% | 3 | 0.4% | 2 | 1.9% | 2 | 0.9% | 146 | 5.6% | <0.001 |
| Other co-morbidity | 867 | 55.6% | 228 | 32.8% | 36 | 34.3% | 82 | 35.7% | 1213 | 46.8% | <0.001 |
| **ER** |  |  |  |  |  |  |  |  |  |  | <0.001 |
| Non-invasive | 141 | 9.0% | 162 | 23.3% | 26 | 24.8% | 57 | 24.8% | 386 | 14.9% |  |
| Negative | 297 | 19.1% | 85 | 12.2% | 18 | 17.1% | 31 | 13.5% | 431 | 16.6% |  |
| Positive | 1105 | 70.9% | 441 | 63.4% | 56 | 53.3% | 131 | 57.0% | 1733 | 66.9% |  |
| Missing | 16 | 1.0% | 8 | 1.2% | 5 | 4.8% | 11 | 4.8% | 40 | 1.5% |  |
| **HER-2** |  |  |  |  |  |  |  |  |  |  | <0.001 |
| Non-invasive | 141 | 9.0% | 162 | 23.3% | 26 | 24.8% | 57 | 24.8% | 386 | 14.9% |  |
| Negative | 1086 | 69.7% | 404 | 58.1% | 61 | 58.1% | 130 | 56.5% | 1681 | 64.9% |  |
| Positive | 272 | 17.5% | 108 | 15.5% | 12 | 11.4% | 28 | 12.2% | 420 | 16.2% |  |
| Missing | 60 | 3.9% | 22 | 3.2% | 6 | 5.7% | 15 | 6.5% | 103 | 4.0% |  |
| **Invasive tumour size (mm)** |  |  |  |  |  |  |  |  |  |  | <0.001 |
| Non-invasive | 141 | 9.0% | 162 | 23.3% | 26 | 24.8% | 57 | 24.8% | 386 | 14.9% |  |
| 0 - 10 | 177 | 11.4% | 134 | 19.3% | 22 | 21.0% | 33 | 14.4% | 366 | 14.1% |  |
| >10 - 20 | 358 | 23.0% | 154 | 22.1% | 17 | 16.2% | 46 | 20.0% | 575 | 22.2% |  |
| >20 - 50 | 671 | 43.0% | 184 | 26.4% | 28 | 26.7% | 63 | 27.4% | 946 | 36.5% |  |
| >50 | 190 | 12.2% | 54 | 7.8% | 9 | 8.6% | 18 | 7.8% | 271 | 10.5% |  |
| Missing | 22 | 1.4% | 8 | 1.2% | 3 | 2.9% | 13 | 5.7% | 46 | 1.8% |  |
| **Lymphovascular invasion** |  |  |  |  |  |  |  |  |  |  | <0.001 |
| Non-invasive | 141 | 9.0% | 162 | 23.3% | 26 | 24.8% | 57 | 24.8% | 386 | 14.9% |  |
| No | 870 | 55.8% | 358 | 51.4% | 49 | 46.7% | 101 | 43.9% | 1378 | 53.2% |  |
| Yes | 435 | 27.9% | 132 | 19.0% | 22 | 21.0% | 46 | 20.0% | 635 | 24.5% |  |
| Missing | 113 | 7.3% | 44 | 6.3% | 8 | 7.6% | 26 | 11.3% | 191 | 7.4% |  |
| **Focality** |  |  |  |  |  |  |  |  |  |  | 0.001 |
| Unifocal disease | 1089 | 69.9% | 442 | 63.5% | 72 | 68.6% | 130 | 56.5% | 1733 | 66.9% |  |
| Multifocal disease | 455 | 29.2% | 249 | 35.8% | 33 | 31.4% | 97 | 42.2% | 834 | 32.2% |  |
| Missing | 15 | 1.0% | 5 | 0.7% | 0 | 0.0% | 3 | 1.3% | 23 | 0.9% |  |
| **Grade of invasive disease** |  |  |  |  |  |  |  |  |  |  | <0.001 |
| Non-invasive | 141 | 9.0% | 162 | 23.3% | 26 | 24.8% | 57 | 24.8% | 386 | 14.9% |  |
| 1 | 98 | 6.3% | 58 | 8.3% | 7 | 6.7% | 16 | 7.0% | 179 | 6.9% |  |
| 2 | 757 | 48.6% | 282 | 40.5% | 47 | 44.8% | 96 | 41.7% | 1182 | 45.6% |  |
| 3 | 543 | 34.8% | 184 | 26.4% | 21 | 20.0% | 50 | 21.7% | 798 | 30.8% |  |
| Missing | 20 | 1.3% | 10 | 1.4% | 4 | 3.8% | 11 | 4.8% | 45 | 1.7% |  |
| **Region** |  |  |  |  |  |  |  |  |  |  | <0.001 |
| London/South East | 274 | 17.6% | 117 | 16.8% | 18 | 17.1% | 74 | 32.2% | 483 | 18.7% |  |
| East | 228 | 14.6% | 90 | 12.9% | 10 | 9.5% | 42 | 18.3% | 370 | 14.3% |  |
| East Midlands | 152 | 9.8% | 41 | 5.9% | 8 | 7.6% | 6 | 2.6% | 207 | 8.0% |  |
| Yorkshire/Humberside | 172 | 11.0% | 71 | 10.2% | 12 | 11.4% | 9 | 3.9% | 264 | 10.2% |  |
| Northern Ireland/Ireland | 54 | 3.5% | 41 | 5.9% | 3 | 2.9% | 5 | 2.2% | 103 | 4.0% |  |
| North | 310 | 19.9% | 137 | 19.7% | 9 | 8.6% | 16 | 7.0% | 472 | 18.2% |  |
| Scotland | 62 | 4.0% | 36 | 5.2% | 30 | 28.6% | 28 | 12.2% | 156 | 6.0% |  |
| Wales/South West | 135 | 8.7% | 36 | 5.2% | 9 | 8.6% | 22 | 9.6% | 202 | 7.8% |  |
| West Midlands | 126 | 8.1% | 40 | 5.8% | 2 | 1.9% | 20 | 8.7% | 188 | 7.3% |  |
| Italy/Egypt | 46 | 3.0% | 87 | 12.5% | 4 | 3.8% | 8 | 3.5% | 145 | 5.6% |  |

Supp Table 2. Percentages where RT is recommended by MDT (or discussed with patient) vs not recommended, stratified by procedure type and T stage or N stage.

| T stage |  | Mastectomy | Implant-based | Pedicled | Free flap | Total |
| --- | --- | --- | --- | --- | --- | --- |
| Tis | % | 12% | 16% | 8% | 11% | 13% |
|  | Of N | 141 | 162 | 26 | 57 | 386 |
| T1-2 | % | 43% | 31% | 37% | 39% | 40% |
|  | Of N | 1206 | 472 | 67 | 142 | 1887 |
| T3 | % | 86% | 94% | 89% | 89% | 88% |
|  | Of N | 190 | 54 | 9 | 18 | 271 |
| Missing | % | 73% | 13% | 33% | 31% | 48% |
|  | Of N | 22 | 8 | 3 | 13 | 46 |
| Total | % | 46% | 32% | 34% | 36% | 41% |
|  | Of N | 1559 | 696 | 105 | 230 | 2590 |
| N stage |  |  |  |  |  |  |
| 0 | % | 24% | 19% | 17% | 26% | 22% |
|  | Of N | 904 | 517 | 71 | 163 | 1655 |
| 1 | % | 71% | 68% | 69% | 60% | 70% |
|  | Of N | 391 | 126 | 16 | 45 | 578 |
| 2 | % | 89% | 93% | 100% | 86% | 90% |
|  | Of N | 250 | 42 | 12 | 14 | 318 |
| Missing | % | 14% | 18% | 17% | 13% | 15% |
|  | Of N | 14 | 11 | 6 | 8 | 39 |
| Total | % | 46% | 32% | 34% | 36% | 41% |
|  | Of N | 1559 | 696 | 105 | 230 | 2590 |

Supp Table 3. Adjusted odds ratios for whether RT is recommended by MDT (or discussed with patient) vs not recommended among a) those undergoing mastectomy only and b) among those undergoing any other type of breast reconstruction.

|  | **Mastectomy (n=1513)** | | | **Non-mastectomy (n=932)** | | |
| --- | --- | --- | --- | --- | --- | --- |
|  | **N (RT rec %)** | **Adjusted odds ratio (95% confidence intervals)** | **P value** | **N (RT rec %)** | **Adjusted odds ratio (95% confidence intervals)** | **P value** |
| **Age** |  |  |  |  |  |  |
| <40 | 66 (75.8%) | 1 |  | 127 (52.0%) | 1 |  |
| 40-49 | 179 (70.4%) | 1.05 (0.57-1.94) | 0.869 | 310 (38.4%) | 0.90 (0.46-1.77) | 0.762 |
| 50-59 | 290 (44.8%) | 0.42 (0.20-0.86) | 0.018 | 292 (30.8%) | 0.64 (0.37-1.12) | 0.116 |
| 60-69 | 395 (40.0%) | 0.46 (0.23-0.93) | 0.03 | 171 (25.7%) | 0.50 (0.30-0.84) | 0.009 |
| 70-79 | 362 (37.3%) | 0.61 (0.31-1.22) | 0.163 | 26 (15.4%) | 0.33 (0.05-2.11) | 0.243 |
| 80+ | 213 (44.1%) | 1.00 (0.39-2.53) | 0.992 | 3 (33.3%) | 0.15 (0.02-0.93) | 0.042 |
| Missing | 8 (50.0%) | 0.54 (0.04-6.70) | 0.631 | 3 (66.7%) | 1.56 (0.19-13.12) | 0.682 |
| **BMI** |  |  |  |  |  |  |
| Underweight | 33 (63.6%) | 3.26 (1.18-8.99) | 0.022 | 11 (36.4%) | 1.01 (0.11-9.13) | 0.99 |
| Normal weight | 441 (46.7%) | 1 |  | 391 (34.8%) | 1 |  |
| Overweight | 447 (45.6%) | 0.87 (0.62-1.22) | 0.427 | 286 (36.7%) | 0.86 (0.41-1.43) | 0.552 |
| Obese | 246 (48.0%) | 0.93 (0.62-1.40) | 0.718 | 131 (37.4%) | 1.01 (0.51-2.00) | 0.987 |
| Severely obese | 222 (45.1%) | 1.00 (0.65-1.55) | 0.997 | 56 (28.6%) | 0.84 (0.38-1.85) | 0.659 |
| Missing | 124 (38.7%) | 0.71 (0.38-1.32) | 0.282 | 57 (28.1%) | 0.92 (0.35-2.43) | 0.867 |
| **IHD** |  |  |  |  |  |  |
| No | 1365 (46.7%) | 1 |  | 909 (35.2%) | 1 |  |
| Yes | 138 (41.3%) | 1.52 (0.89-2.59) | 0.125 | 7 (71.4%) | 9.06 (1.23-66.93) | 0.031 |
| Missing | 10(20.0%) | 0.37 (0.08-1.81) | 0.221 | 16 (6.3%) | 0.03 (0.00-0.24) | 0.001 |
| **Diabetes** |  |  |  |  |  |  |
| No | 1290 (46.4%) | 1 |  | 866 (34.9%) | 1 |  |
| Yes | 187 (44.9%) | 1.30 (0.83-2.04) | 0.252 | 42 (40.5%) | 1.08 (0.31-3.75) | 0.905 |
| Missing | 36 (38.9%) | 0.39 (0.15-1.02) | 0.054 | 24 (29.2%) | 1.22 (0.29-5.15) | 0.784 |
| **Other comorbidities** |  |  |  |  |  |  |
| No | 643 (51.5%) | 1 |  | 607 (36.6%) | 1 |  |
| Yes | 861 (42.0%) | 1.01 (0.68-1.50) | 0.943 | 314 (32.5%) | 0.93 (0.60-1.46) | 0.764 |
| Missing | 9 (44.4%) | 0.81 (0.06-11.52) | 0.874 | 11 (18.2%) | 0.33 (0.05-1.95) | 0.221 |
| **Smoking status** |  |  |  |  |  |  |
| Non-smoker | 1073 (45.8%) | 1 |  | 686 (34.1%) | 1 |  |
| Ex-smoker | 237 (46.4%) | 1.12 (0.74-1.70) | 0.601 | 152 (35.5%) | 1.04 (0.59-1.83) | 0.896 |
| Current smoker | 174 (48.3%) | 1.03 (0.68-1.54) | 0.902 | 78 (38.5%) | 1.35 (0.68-2.68) | 0.393 |
| Missing | 29 (41.4%) | 1.60 (0.51-5.03) | 0.421 | 16 (50.0%) | 2.39 (0.73-7.89) | 0.151 |
| **Chemotherapy** |  |  |  |  |  |  |
| Not recommended | 685 (23.1%) | 1 |  | 428 (14.0%) | 1 |  |
| Recommended | 372 (65.3%) | 4.07 (2.57-6.47) | <0.001 | 195 (60.0%) | 5.16 (2.79-9.54) | <0.001 |
| For discussion | 135 (56.3%) | 5.28 (3.17-8.79) | <0.001 | 45 (20.0%) | 1.37 (0.61-3.06) | 0.442 |
| For oncotype DX | 92 (28.3%) | 1.76 (1.04-2.96) | 0.034 | 86 (18.6%) | 1.32 (0.47-3.69) | 0.599 |
| Already received | 229 (84.7%) | 24.65 (12.57-48.35) | <0.001 | 178 (69.7%) | 9.36 (4.95-17.68) | <0.001 |
| **ASA grade** |  |  |  |  |  |  |
| 1 | 329 (52.0%) | 1 |  | 374 (34.8%) | 1 |  |
| 2 | 887 (45.2%) | 1.01 (0.71-1.46) | 0.938 | 529 (34.6%) | 1.02 (0.66-1.56) | 0.942 |
| 3 | 283 (41.3%) | 0.88 (0.52-1.51) | 0.649 | 27 (44.4%) | 0.96 (0.25-3.66) | 0.951 |
| 4 | 6 (50.0%) | 0.77 (0.13-4.64) | 0.777 | 0 (0.0%) | NA | NA |
| Missing | 8 (62.5%) | 2.26 (0.77-6.59) | 0.136 | 2 (50.0%) | 0.62 (0.12-3.26) | 0.583 |
| **ER** |  |  |  |  |  |  |
| Non-invasive | 140 (12.1%) | 0.84 (0.34-2.08) | 0.699 | 228 (14.5%) | 1.25 (0.54-2.87) | 0.602 |
| Negative | 285 (53.3%) | 1 |  | 124 (45.2%) | 1 |  |
| Positive | 1072 (48.7%) | 1.41 (0.92-2.15) | 0.114 | 566 (41.7%) | 0.96 (0.58-1.57) | 0.861 |
| Missing | 16 (37.5%) | 0.29 (0.02-3.85) | 0.346 | 14 (7.1%) | 0.00 (0.00-0.04) | <0.001 |
| **HER-2** |  |  |  |  |  |  |
| Non-invasive | 140 (12.1%) | NA | NA | 228 (14.5%) | NA | NA |
| Negative | 1050 (48.8%) | 1 |  | 544 (40.6%) | 1 |  |
| Positive | 264 (54.9%) | 0.77 (0.52-1.14) | 0.185 | 128 (50.0%) | 0.87 (0.53-1.43) | 0.578 |
| Missing | 59 (39.0%) | 0.77 (0.42-1.41) | 0.394 | 32 (25.0%) | 1.81 (0.52-6.33) | 0.351 |
| **Invasive tumour size (mm)** |  |  |  |  |  |  |
| Non-invasive | 140 (12.1%) | NA | NA | 228 (14.5%) | NA | NA |
| 0 - 10 | 171 (38.0%) | 1 |  | 169 (32.0%) | 1 |  |
| >10 - 20 | 346 (28.9%) | 0.65 (0.37-1.11) | 0.115 | 188 (27.7%) | 0.75 (0.41-1.37) | 0.351 |
| >20 - 50 | 645 (52.1%) | 1.42 (0.88-2.27) | 0.147 | 256 (43.4%) | 1.12 (0.66-1.92) | 0.674 |
| >50 | 189 (86.2%) | 11.60 (5.74-23.43) | <0.001 | 77 (93.5%) | 51.82 (9.92-270.52) | <0.001 |
| Missing | 22 (72.7%) | 80.36 (1.71-3771.75) | 0.025 | 14 (28.6%) | 2.30 (0.58-9.08) | 0.233 |
| **Lymphovascular invasion** |  |  |  |  |  |  |
| Non-invasive | 140 (12.1%) | NA | NA | 228 (14.5%) | NA | NA |
| No | 848 (40.2%) | 1 |  | 455 (32.3%) | 1 |  |
| Yes | 428 (67.5%) | 1.35 (0.90-2.02) | 0.151 | 188 (61.7%) | 1.28 (0.70-2.34) | 0.431 |
| Missing | 97 (51.6%) | 1.32 (0.78-2.21) | 0.297 | 61 (49.2%) | 1.93 (0.78-4.74) | 0.154 |
| **Lymph nodes** |  |  |  |  |  |  |
| N0 | 884 (23.9%) | 1 |  | 673 (22.0%) | 1 |  |
| N1 | 377 (72.4%) | 5.37 (3.72-7.75) | <0.001 | 174 (68.4%) | 4.57 (2.42-8.65) | <0.001 |
| N2 | 238 (88.7%) | 13.08 (7.52-22.76) | <0.001 | 60 (91.7%) | 17.10 (4.89-59.77) | <0.001 |
| Missing | 14 (14.3%) | 0.18 (0.02-1.72) | 0.138 | 25 (16.0%) | 0.42 (0.13-1.37) | 0.152 |
| **Focality** |  |  |  |  |  |  |
| Unifocal disease | 1056 (43.5%) | 1 |  | 585 (33.0%) | 1 |  |
| Multifocal disease | 442 (52.5%) | 1.40 (0.95-2.07) | 0.09 | 339 (38.9%) | 1.16 (0.81-1.66) | 0.414 |
| Missing | 15 (40.0%) | 0.86 (0.21-3.49) | 0.836 | 8 (12.5%) | 4.76 (0.34-67.47) | 0.249 |
| **Grade of invasive disease** |  |  |  |  |  |  |
| Non-invasive | 140 (12.1%) | NA | NA | 228 (14.5%) | NA | NA |
| 1 | 98 (33.7%) | 1.31 (0.76-2.23) | 0.328 | 76 (17.1%) | 1.12 (0.62-2.03) | 0.704 |
| 2 | 737 (48.4%) | 1 |  | 390 (41.0%) | 1 |  |
| 3 | 518 (54.6%) | 0.94 (0.61-1.43) | 0.758 | 223 (52.0%) | 1.17 (0.71-1.93) | 0.537 |
| Missing | 20 (35.0%) | 0.22 (0.03-1.92) | 0.172 | 15 (26.7%) | 7.38 (1.30-41.86) | 0.024 |
| **Region** |  |  |  |  |  |  |
| London/South East | 274 (52.9%) | 1 |  | 209 (39.2%) | 1 |  |
| East | 228 (48.3%) | 0.84 (0.46-1.52) | 0.562 | 142 (38.7%) | 0.71 (0.25-1.99) | 0.512 |
| East Midlands | 152 (49.3%) | 0.93 (0.46-1.89) | 0.837 | 55 (23.6%) | 0.45 (0.17-1.20) | 0.11 |
| Yorkshire/Humberside | 172 (37.2%) | 0.49 (0.26-0.91) | 0.024 | 92 (23.9%) | 0.31 (0.11-0.83) | 0.02 |
| Northern Ireland/Ireland | 54 (51.9%) | 0.30 (0.15-0.60) | 0.001 | 49 (46.9%) | 1.85 (0.65-5.29) | 0.251 |
| North | 310 (40.7%) | 0.43 (0.22-0.84) | 0.014 | 162 (24.7%) | 0.43 (0.17-1.05) | 0.065 |
| Scotland | 62 (43.6%) | 0.33 (0.17-0.64) | 0.001 | 94 (47.9%) | 1.32 (0.46-3.80) | 0.613 |
| Wales/South West | 135 (46.7%) | 0.74 (0.37-1.47) | 0.387 | 67 (34.3%) | 1.81 (0.61-5.36) | 0.282 |
| West Midlands | 126 (46.8%) | 0.51 (0.28-0.95) | 0.034 | 62 (37.1%) | 1.02 (0.23-4.50) | 0.974 |
