# Supplementary material for: Patterns of Postmastectomy Radiotherapy in Immediate Breast Reconstruction—Results From the iBRA‐2 Cohort Study
Source: Int J Breast Cancer. 2026 May 18;2026:5902426. doi: 10.1155/ijbc/5902426 (PMC13181802; doi:10.1155/ijbc/5902426)
Supplement: Supplementary file 2 — Supporting Information 2 Figure S1: Scatter plot of implant‐only reconstruction (IBR) percentages and postmastectomy radiotherapy (PMRT) recommendation percentages (n = 2381, excluding centres outside the UK and Ireland). [file IJBC-2026-5902426-s002.docx]

Supp Figure 1. Scatter plot of implant-based reconstruction (IBR) percentages and post-mastectomy radiotherapy (PMRT) recommendation percentages (n=2,381, excluding centres outside UK and Ireland).

*r is Pearson’s r statistic and associated p-value (p)
